# Isoform-specific interactions of the von Hippel-Lindau tumor suppressor protein

**DOI:** 10.1038/srep12605

**Published:** 2015-07-27

**Authors:** Giovanni Minervini, Gabriella M. Mazzotta, Alessandro Masiero, Elena Sartori, Samantha Corrà, Emilio Potenza, Rodolfo Costa, Silvio C. E. Tosatto

**Affiliations:** 1 Department of Biomedical Sciences, University of Padova; 2 Department of Biology, University of Padova; 3 CNR Institute of Neuroscience, Padova, Italy

## Abstract

Deregulation of the von Hippel-Lindau tumor suppressor protein (pVHL) is considered one of the main causes for malignant renal clear-cell carcinoma (ccRCC) insurgence. In human, pVHL exists in two isoforms, pVHL19 and pVHL30 respectively, displaying comparable tumor suppressor abilities. Mutations of the p53 tumor suppressor gene have been also correlated with ccRCC insurgence and ineffectiveness of treatment. A recent proteomic analysis linked full length pVHL30 with p53 pathway regulation through complex formation with the p14ARF oncosuppressor. The alternatively spliced pVHL19, missing the first 53 residues, lacks this interaction and suggests an asymmetric function of the two pVHL isoforms. Here, we present an integrative bioinformatics and experimental characterization of the pVHL oncosuppressor isoforms. Predictions of the pVHL30 N-terminus three-dimensional structure suggest that it may exist as an ensemble of structured and disordered forms. The results were used to guide Yeast two hybrid experiments to highlight isoform-specific binding properties. We observed that the physical pVHL/p14ARF interaction is specifically mediated by the 53 residue long pVHL30 N-terminal region, suggesting that this N-terminus acts as a further pVHL interaction interface. Of note, we also observed that the shorter pVHL19 isoform shows an unexpected high tendency to form homodimers, suggesting an additional isoform-specific binding specialization.

Renal clear-cell carcinoma (ccRCC) is a renal cortical tumor characterized by malignant cells with clear cytoplasm, making up about 75% of kidney cancers^1^. Generally, ccRCC is described as a heterogeneous disease presenting well distinct subtypes harboring severe cytogenetic aberrations^2^. Moreover, ccRCC is frequently resistant to both chemotherapy and radiotherapy, presenting diffuse tissue hypoxia^3^. Poor diagnosis and resistance to treatment are associated to the deregulation of hypoxia inducible factor HIF-1α, a key transcription factor regulating the expression of over 200 genes^4^. In the last two decades, disorders of the p53 tumor suppressor gene have been correlated with ccRCC insurgence, with presence of p53 mutants in neoplastic tissues frequently associated with reduced or null responsiveness to treatment^5^,^6^,^7^. However, extensive sequence analysis in patients demonstrated that p53 is rarely mutated in ccRCC, rather suggesting that the p53 pathway itself might be compromised^7^. Interaction between p53 and HIF-1α was actively investigated, leading to the discovery of a strong correlation between the latter and ccRCC malignant evolution^8^. ccRCC is also one of the most common manifestations of von Hippel Lindau (VHL) syndrome, where inactivation of the pVHL tumor suppressor, a component of the E3 ubiquitin ligase complex targeting HIF-1α for degradation, leads to development of lethal ccRCC in patients^9^. The VHL tumor suppressor gene contains 3 exons, encoding a protein of 213 amino acid residues, known as pVHL30^10^. A second isoform, termed pVHL19, is also expressed in human. pVHL19 arises from an alternate translation initiation site within the VHL open reading frame yielding a 160 residue protein missing residues 1 to 53^11^. Both isoforms display tumor suppressor abilities^12^, inhibiting cancer development when a wild type copy is reintroduced in ccRCC cells^13^. In 2006, a functional correlation between p53 and pVHL was demonstrated^14^, providing an important insight into the molecular mechanisms by which deregulation of both tumor suppressors yields cancer. Neoplastic cells share a metabolism demanding high oxygen levels and high proliferative rates. Of note, pVHL is known to regulate angiogenesis and mediate cellular adaptation to hypoxia, whereas p53 promotes hypoxia-inducible apoptosis^15^. That p53 degradation is mediated by the E3 ubiquitin-protein ligase MDM2 is currently well known^16^ and a parallelism between the p53 and HIF-1α degradation strategies was suggested in the literature^15^,^17^. p53 is also regulated by the p14ARF protein, an alternative reading frame product of the CDKN2A locus also encoding for the tumor suppressor p16ink4a^18^. p14ARF acts by inhibiting nuclear MDM2, yielding an up-regulation of p53 transcriptional response^19^. A proteomic co-immunoprecipitation assay recently detected an interaction between pVHL and p14ARF, suggesting a deeper connection between the two tumor suppressor pathways^20^. Of note, Shiio and coworkers reported that p14RF binds the mainly cytoplasmic pVHL30 isoform, while interaction with nuclear pVHL19 was not detected^20^. The two isoforms differ by a N-terminal tail (pVHL-N) present in the former, which was suspected to be responsible for the different subcellular localization^11^. Clues for different HIF-1α-independent functions of pVHL were proposed in the literature^12^ but the molecular details are not completely understood. Here, we aim to dissect the function the pVHL N-terminal tail in different regulation pathways using an integrative bioinformatics and Yeast two-hybrid interaction assays.

## Methods

### Bioinformatics characterization

Structural investigation was conducted using bioinformatics web tools starting from the UniProt database^21^. Accession codes: P40337 and Q8N726 for human pVHL and p14ARF, respectively) were used for the sequences. Predictions of intrinsic disorder were performed using MobiDB^22^, while manual inspection was used to predict the repeat behavior. CSpritz^23^ was used to annotate the secondary structure propensity of disordered segments. Presence of eukaryotic functional sites were predicted with ELM^24^. The multiple sequences alignment was generated from 43 pVHL orthologs selected by OMA Browser^25^ aligned using ClustalW^26^. OMA was used to reduce the indetermination connected with manual sequence selection. pVHL exists in two isoforms in human and many other organisms. In addition, many species (i.e. zebrafish and human) present an accessory paralog termed “VHL-like”, which adds complexity to manual sequence selection. The final alignment has been manually refined at the variable N-terminus using Jalview^27^. The OMA identifiers used to build the alignment are: HUMAN19991, ANOGA04345, CHICK03110, DANRE22808, MOUSE18976, XENTR07973, APIME04810, RATNO15510, TAKRU07960, BOVIN10135, PANTR11972, LOXAF14953, RABIT12727, DASNO11236, ECHTE06585, GASAC02310, ORYLA13738, FELCA02104, OTOGA15552, SPETR08654, MYOLU15825, CAVPO03478, MICMU12561, OCHPR05038, PONAB10576, HELRO20977, LOTGI22650, TURTR10344, PTEVA12606, PROCA00524, DIPOR02505, GORGO13436, ANOCA16022, PIGXX01635, CALJA07552, AILME06784, NOMLE02564, GADMO19692, LATCH19084, ORENI11836, PELSI15488, MUSPF19501, XIPMA15655, FICAL04046, LEPOC13881, ASTMX09426. A graphical representation of the alignment is reported in Figure S1. The structure of the pVHL N-terminus, residues 1 to 53, was predicted using the Rosetta *ab initio* prediction software^28^ using a previously described protocol optimized for intrinsically disordered proteins^29^. A total of 25,000 decoys were generated and clustered, with the ten most frequent models visually inspected with Chimera^30^.

### Nucleotide sequences, primers and extraction protocol

Positions reflect the nucleotide location in the NCBI Reference Sequences: NP_000542.1 (VHL) and NM_058195.3 (p14ARF). Primer nomenclature, positions, directions and sequences were set as described in Table 1. Protein extracts were obtained as in^31^, subjected to SDS-Page (NuPAGE- Invitrogen®) and probed with anti-MYC (CLONTECH, 1:5000) and anti-HA (SIGMA, 1:5000) antibodies. The expression level of the fusion proteins was then quantified with the Image J software (available at http://rsb.info.nih.gov/ij; developed by Wayne Rasband, National Institutes of Health).

### Yeast two-hybrid system

All experiments were performed using the Matchmaker® Gold Yeast Two-Hybrid System (Clontech, Mountain View, CA, USA), in the Y2HGold strain, using MEL1 (encoding alpha-galactosidase) as reporter gene. The full-length hVHL coding sequence was amplified using Phusion® High-Fidelity DNA Polymerase (New England Biolabs, Ipswich, MA), from Addgene plasmid 20790 (Addgene, Cambridge, MA, USA) and cloned alternatively in pGBKT7 DNA-BD or pGADT7-AD cloning vectors. Primers were specifically designed for a “one-step PCR cloning” with the In-Fusion® Advantage PCR Cloning Kit (Clontech, Mountain View, CA, USA). All constructs with the different pVHL fragments were obtained with the same strategy. The full-length p14ARF coding sequence was amplified from cDNA retro-transcribed from the Universal Human Reference RNA, a pool of total RNA from 10 human cell lines (Agilent Technologies, Santa Clara, CA, USA) and cloned in a pGADT7-AD cloning vector. All constructs were fully sequenced to assess the in-frame insertion of the cDNA and to control for unwanted mutations. The reliable expression of bait and fusions was assessed by immunoblot (Figure S2). The catalytic activity of alpha-galactosidase was monitored colorimetrically by measuring the rate of hydrolysis of the chromogenic substrate, p-nitrophenyl-alpha-D-galactopyranoside. One of the products of this reaction, p-nitrophenol, displays a strong absorption band at 410 nm. Quantification of alpha-galactosidase activity was performed in liquid culture as in^32^, with the following modifications. Transformed yeast cells, cultured in selective medium to an OD = 0.7, were pelleted in a bench centrifuge at 16000 rcf for 5 min. 200 ml of medium were transferred to 600 ml of Assay Buffer (0.33 M sodium acetate pH 4.5; 10 mg/ml p-nitrophenyl-alpha-D-galactopyranoside), incubated at 29 °C for 16 hours, and the reaction was stopped through addition of 200 ml of 2M Na_2_CO_3_. Alpha-galactosidase activity was calculated as: alpha-galactosidase units = 1,000 × OD410/(t × V × OD600) where: t = time (in minutes) of incubation, V = volume of culture used in the assay (ml), OD410 = absorbance by p-nitrophenol, OD600 = cell density at the start of the assay. Seven to ten independent clones were analyzed for each construct and the measured activity was related to the expression level of the fusion protein involved. Each experiment was performed in triplicate. Statistic analysis was performed with Graphpad Prism v4 using one-way ANOVA followed by Tukey’s multiple comparisons test.

## Results And Discussion

### pVHL N-terminal tail as molecular hook

**Figure 1:**
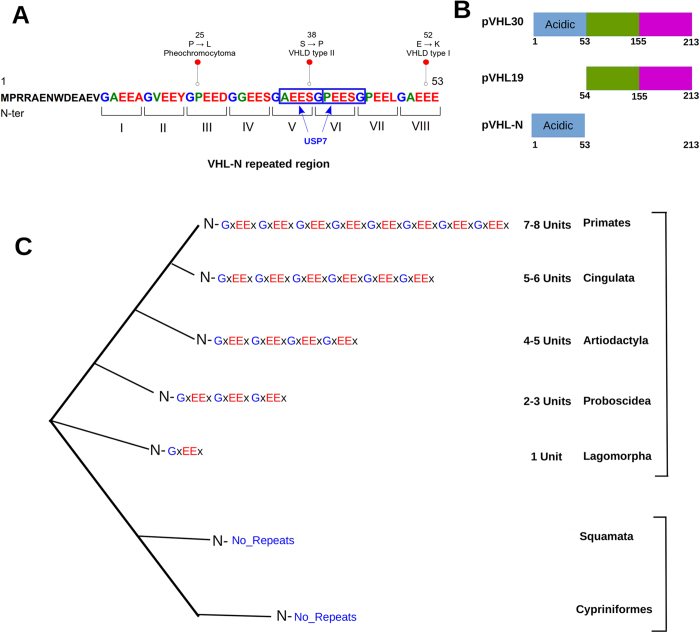
Bioinformatics characterization of human pVHL N-terminus. (**A**) Roman numerals are used for repeats, while mutations promoting cancer insurgence are presented with red dots. Blue boxes are used for predicted USP7 binding sites. (**B**) Schematic representation of pVHL isoforms. The N-terminal tail is presented in light blue, while green and purple are used for the β- and α-domain forming pVHL, respectively. A multiple sequence alignment of pVHL is presented in Figure S2. (**C**) Simplification of pVHL-N sequences found in different animals. Repeat units were only found in mammals, with their number varying among species.

**Figure 2:**
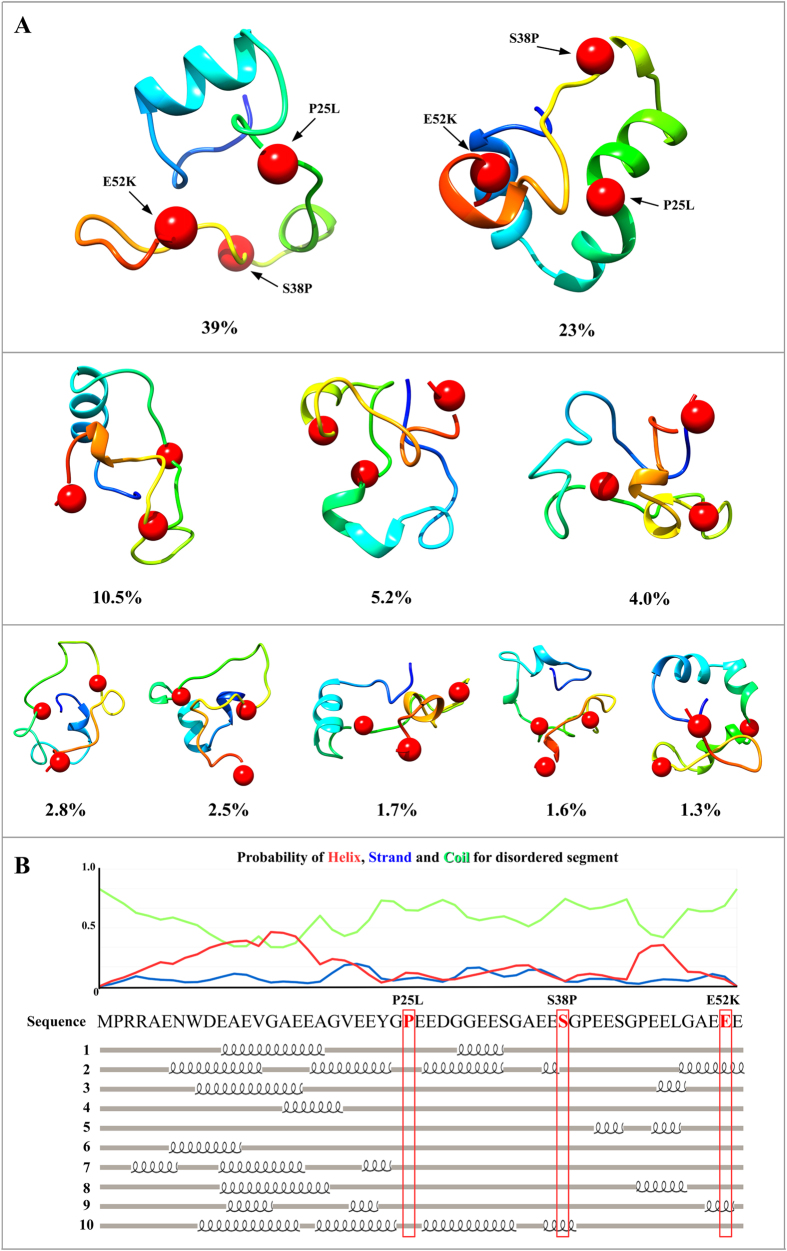
Top ten *ab initio* models of pVHL-N. (**A**) The ten best models of the pVHL30 N-terminal tail, ordered by relative frequency. The models were extracted by clustering 25,000 decoys. Red spheres represent the position of mutations found in VHL patients. (**B**) pVHL-N secondary structure propensities from CSpritz^23^ are shown. Intrinsic disorder predictions for pVHL-N from MobiDB^22^, with the orange line representing a consensus of different methods (i.e. full disorder). pVHL-N secondary structures, calculated starting from the three-dimensional models, are shown together with the sequence. Red boxes are used to highlight the P25L, S38P, E52K mutation positions. For each line, the percentage of disordered residues is presented on the left side.

A bioinformatics analysis was performed to better understand the role of pVHL-N. The computational characterization showed that the N-terminus of pVHL30 is both repeated in sequence and intrinsically disordered. Indeed, the region is formed by eight short repeat units presenting the “*GxEEx*” motif, with x representing less conserved amino acids (Fig. 1). Intrinsic disorder prediction suggested that the region is prone to be unstructured, coherent with data in the literature^33^. Indeed, pVHL-N was not solved in the x-ray structure (PDB code 1LM8), suggesting a different structural organization of the region when compared to the rest of the protein. Intrinsic protein disorder is commonly associated with protein-protein interactions^34^, while repeat proteins have assumed a relevant position in cancer biology during the last years^35^,^36^. Considering both phenomena, we suggest that the pVHL-N region acts as a molecular adapter, where folding is strongly associated to the binding partner. To address this specific behavior, we first searched for functional sites in this region. We found two predicted USP7 binding motifs (residues 35–44, see Fig. 1), a deubiquitinating enzyme co-localizing with p53 and Mdm2 and involved in their regulation^37^. While the USP7 motifs are quite generic and may be prone to overprediction, this finding is nevertheless fascinating as it may suggest a more extensive cross-talk between the pVHL and p53 oncosuppressors. Second, we performed an *ab initio* structure prediction of the first 53 residues, in order to investigate the fold adopted by pVHL-N. Ten different models were generated (Fig. 2) by clustering 25,000 decoy structures. The predictions appear to confirm the intrinsically unfolded nature of this region, with four out of five models assuming a disordered conformation. The most populated conformational cluster, accounting for approximately 39% of the decoys, is mostly disordered (Fig. 2). However, the second model (23%) presents a more compact structure, with well structured and repeated α-helix elements (Fig. 2). A certain propensity to form short α-helices was found in all generated models in a region between residues 9 and 18, immediately before the “*GxEEx*” repeat, in particular for human. This finding suggests that the pseudo-folded segments may help pVHL establish protein-protein interactions, acting as a nucleation element driving the N-terminus folding process. In particular, model 2 (Fig. 2) seems to support this hypothesis. The model is mostly organized in an all-α structure, with a general fold similar to the three-helix bundle fold. This fold, typical in actin binding proteins, is known to be one of the smallest and fastest cooperatively folding structural domains^38^. We hypothesize that the pVHL-N is intrinsically disordered when unbound and quickly reverts to a folded state when interacting with binding partners. Of note, some experimental evidence suggested a role for pVHL in the regulation of actin assembly and cell motility inhibition^39^. In other words, due to its ambivalent repeated and disordered nature, we speculate that pVHL-N could be a region that folds upon binding in order to maximize molecular plasticity of pVHL. This phenomenon is known to be also occurring in other cancer related proteins^40^. Mutations in this region promoting cancer insurgence are described in the literature^41^ (Fig. 1A). In particular, P25L, S38P, E52K were found in pheochromocytoma, in VHL disease type II and VHL disease type I, respectively (source Uniprot database^21^). The two best models, accounting for ca. 62% of all generated structures were used to characterize pVHL-N specific mutations (Fig. 2). We found that the mutations affect regions prone to form short α-helix elements, in particular P25L is localized between two α-helices conferring a characteristic rigid “V” shape to the N-terminus. According to the severity of the mutant phenotype, we suggest that P25L may interfere with the three-helix bundle fold, destabilizing the relative orientation of the α-helices. The S38P mutation affects a prevalently unfolded loop and sequence analysis revealed this residue to be included in a putative USP7 binding motif. Insertion of proline disrupts this putative signal, suggesting an explanation for the pathological effect. Our structure predictions also suggested that the substitution may limit pVHL-N flexibility and interfe with its protein-protein binding propensity. Finally the E52K mutation inserts a positive charge in a negative triplet formed by the glutamic acid residues 51–53. Considering the mainly acidic pVHL-N composition, this mutation should promote an irregular salt-bridge bond, reducing conformational flexibility and stabilizing an unfolded knot promoting a malignant phenotype.

### pVHL30 N-terminal tail is responsible for p14ARF-pVHL interaction

**Figure 3:**
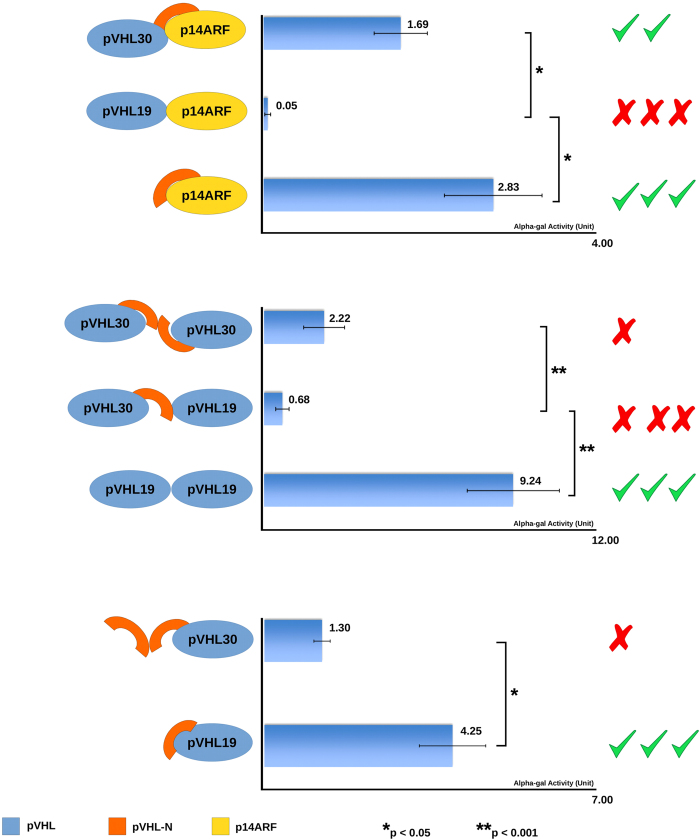
pVHL interactions in the yeast two-hybrid system. Different combinations of pVHL, full-length or fragments, were tested for binding to p14ARF, dimerization and/or intra-molecular interactions. Graphical representation of different tested conditions is presented in the picture. Alpha-galactosidase activity is reported for each fusion. Mean ± SEM (standard error of the mean) of seven to ten independent clones for each fusion is shown. See methods for details.

Although the importance of pVHL in ccRCC outcome has long been recognized, the exclusive molecular function of the two isoforms remains poorly understood. Recently, Shiio and coworkers demonstrated a functional differentiation between human isoforms of pVHL^20^. In particular, a proteomic co-immunoprecipitation assay showed that pVHL30, but not pVHL19, is found in a complex with the oncosuppressor p14ARF^20^. The result is quite impressive, as it is the first evidence of functional differentiation between pVHL proteins, at least in human. pVHL-N is a short region of 53 amino acids found in human and other higher primates with little or no conservation, in other organisms^42^. Moreover, due to relatively low evolutionary conservation (Fig. 1) pVHL-N is generally considered of lesser importance for pVHL tumor suppressor function than the rest of the protein^42^. To determine a pVHL-N specific role, we studied its interaction properties in a Yeast two-hybrid system. First of all, we have tested the two different pVHL isoforms (pVHL30 and pVHL19) and the pVHL-N region as baits and challenged them against p14ARF as prey (Fig. 3A). We have observed that pVHL30, but not pVHL19, is able to bind p14ARF, confirming previous data^20^, and that pVHL-N is responsible for the interaction (Fig. 3A). These results confirmed that the interaction between the two oncosuppressors is specifically sustained by pVHL-N and does not depend on interactions with other proteins. pVHL is known to interact with more than 200 different interactors^43^ forming a vast and complex pathway^44^. In a previous work, we proposed three different binding areas for pVHL, with most of the protein surface involved in protein-protein interactions^45^. The results presented here show a fourth binding area at the N-terminal region. According to the large number of pVHL known interactors, we speculate that other proteins may specifically interact with pVHL-N. In other words, we suggest that pVHL30 functional specialization may go beyond the sole p14ARF interaction.

### pVHL19 forms a homodimer

In 2006, Ohh and coworkers showed that pVHL forms self-associated complexes *in vivo*, while the same homo-association was not found *in vitro*^46^ and the authors concluded that functional disparity was perhaps related with certain modification(s)/processing not easily reproducible by the *in vitro* condition^46^. In order to address this specific pVHL behavior, we tested the involvement of pVHL-N in pVHL homodimeric association. Our *in silico* analysis showed that the N-terminal region contains eight copies of an acidic pentamer repeat (Fig. 1C). On the other hand, analysis of the pVHL/HIF-1α binding interface revealed a pVHL mainly exposing positively charged residues. The finding suggested a possible interaction between two pVHL30 with the N-terminal region acting as a molecular zipper. We then studied the specific dimerization properties of pVHL analyzing the different protein domains (Fig. 3B). We observed a certain homodimeric interaction for pVHL30, which does not seem to be due to the N-terminal region, as it is reduced, although not significantly, when pVHL-N is tested against pVHL30 (Fig. 3C). On the contrary, the pVHL19 isoform shows an unexpected high tendency to form homodimers (Fig. 3B). This isoform also interacts with the N-terminal region, but not with pVHL30 (Fig. 3B,C). Considering this previously unreported finding, we suggest that pVHL-N actively inhibits homodimeric association through binding competition for the same area. The so-called β-domain of pVHL is well known to interact with HIF-1α^47^. Here we demonstrated that the same area is also responsible for pVHL19 homodimeric association. On the other hand, lack of significant interactions between two pVHL30 or pVHL30 and pVHL-N rejected the idea of a N-terminal molecular zipper. Of note, a suspected pVHL homodimeric association was previously reported in the literature as a prerequisite for fibronectin matrix assembly^48^, while pVHL19 was shown to specifically interact with collagen^20^. Coupling this finding with data presented here, we propose a pVHL19 homodimeric complex as the second functional specialization observed for pVHL isoforms. Due to the importance pVHL assumes in ccRCC outcome, we believe that both the N-terminal region and homodimeric pVHL19 complex may be used as target for drug development (i.e. N-terminal mimetic peptide) capable to interfere with malignant cancer progression.

## Conclusions

**Figure 4:**
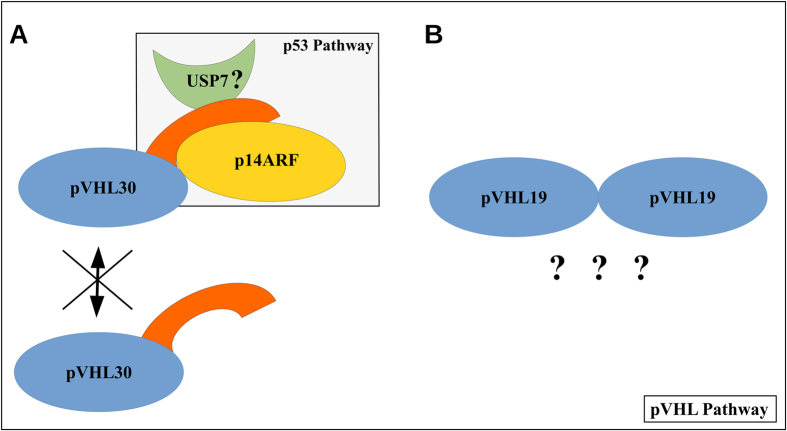
Schematic overview of the main findings.

While the association between pVHL mutations and benign lesions in VHL patients is well known and experimentally validated, the molecular mechanism yielding malignant progression remains unclear. Considering human cancer, in particular ccRCC, as a multistep process, the precise regulation typically active in healthy tissue should be bypassed to allow malignant progression. Cancer requires a constant blood supply to obtain the nutrients and oxygen it needs to grow and pVHL inactivation represents an early deregulative step. A mutated pVHL isoform promoting deregulation of different pathway regulators, e.g. p53, may represent the molecular switch modulating ccRCC outcome. The results reported in this study indicate that the pVHL19 and pVHL30 isoforms have different binding properties which may represent different pVHL tumor suppressor activities (Fig. 4). The idea of isoform-specific functional specialization is emerging from the literature as suggested by experimental evidence^49^,^50^. However, it is unclear how these isoforms are connected with cancer outcome. Here, we showed that the pVHL30 N-terminal tail may act as a molecular hook. We demonstrated that the isolated tail alone is able to drive interaction with p14ARF, connecting this specific pVHL isoform with the p53 pathway and suggesting that a part of the phenotypic variability observed in VHL disease may be due to mutations affecting proteins involved in this oncosuppressor pathway. On the other hand, we demonstrated that only pVHL19 forms homodimers, reinforcing the hypothesis for the two pVHL isoforms to be brothers but not twins. Our results suggest that isoform-specific functions may have a role in malignancy of VHL syndrome manifestations. Data presented in this study can give a useful insight on the basic structural properties of pVHL isoforms and their specific properties.

## Additional Information

**How to cite this article**: Minervini, G. *et al.* Isoform-specific interactions of the von Hippel-Lindau tumor suppressor protein. *Sci. Rep.* **5**, 12605; doi: 10.1038/srep12605 (2015).

## Supplementary Material

Supplementary Information

## Figures and Tables

**Table 1:** Nucleotide sequences and position of primers used.

Primer	Position	Direction	Sequence (5’→3’)
*pGB_VHL30_F*	1–21	F	ggaggacctg*catatg***ATGCCCCGGAGGGCGGAGAAC**
*pGB_VHL30_R*	619–642	R	gcatccccgg*gaattc***TCAATCTCCCATCCGTTGATGTC**
*pGAD_VHL30_F*	1–21	F	agattacgct*catatg***ATGCCCCGGAGGGCGGAGAAC**
*pGAD_VHL30_R*	619–642	R	cacccgggtg*gaattc***TCAATCTCCCATCCGTTGATGTGC**
*pGB_VHL19_F*	160–171	F	ggaggacctg*catatg***ATGGAGGCCGGGCGGCCGCGG**
*pGAD_VHL19_F*	160–171	F	agattacgct*catatg***ATGGAGGCCGGGCGGCCGCGG**
*pGB_VHL_N_R*	139–159	R	gcatccccgg*gaattc***TCACTCCTCCTCGGCGCCCAG**
*pGAD_p14ARF_F*	1–23	F	agattacgct*catatg***ATGGTGCGCAGGTTCTTGGTGAC**
*pGAD_p14ARF_R*	376–399	R	cacccgggtg*gaattc***TCAGCCAGGTCCACGGGCAGACGG**

Position reflects nucleotide location in NCBI Reference Sequence: NP_000542.1 (VHL) and NM_058195.3 (p14ARF).
