# Supplementary material for: Isoform-specific interactions of the von Hippel-Lindau tumor suppressor protein
Source: Sci Rep. 2015 Jul 27;5:12605. doi: 10.1038/srep12605 (PMC4515828; doi:10.1038/srep12605)
Supplement: Supplementary Information [file srep12605-s1.doc]

**Isoform-specific interactions of the von Hippel-Lindau tumor suppressor protein**

Giovanni Minervini^1a^, Gabriella M Mazzotta^2a^, Alessandro Masiero^1^, Elena Sartori^2^, Samantha Corrà^2^, Emilio Potenza^1^ , Rodolfo Costa^2^, Silvio CE Tosatto^1^*.

* *Corresponding Author*.

^a^ *These authors contributed equally to this work*

**Affiliation:** ^1^*Department of Biomedical Sciences, University of Padova, Italy, ^2^Department of Biology, University of Padova, Italy.*

**Supplementary materials**


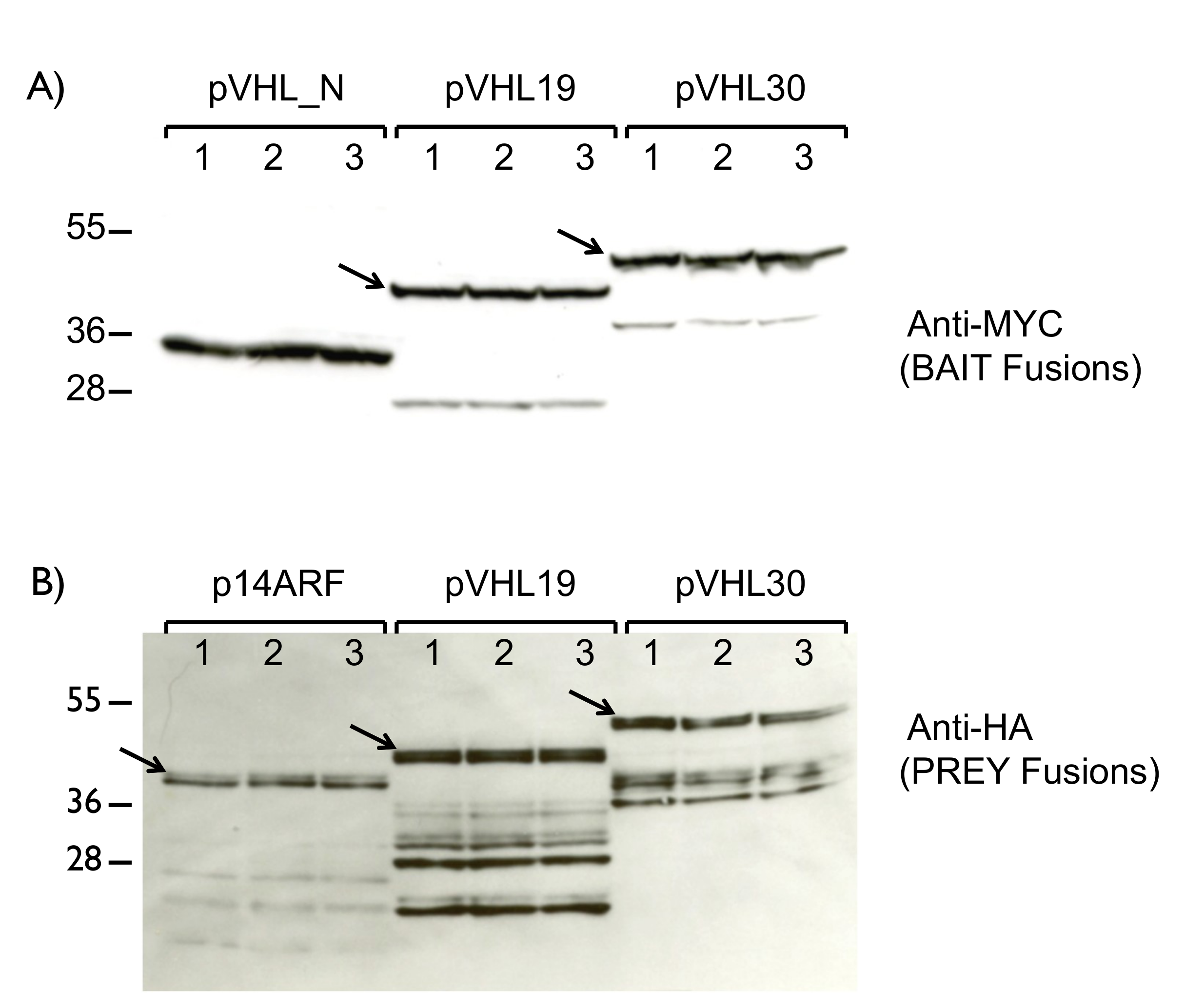


**Figure S1. Western blot analysis of independent yeast clones for BAIT (A) and PREY (B) fusions expressing different fragments of pVHL or p14ARF.**

Three independent yeast clones for each fusion probed with anti-MYC and anti-HA antibodies, for the detection of bait and prey fusions, respectively. Arrows indicate the signals corresponding to the fusions. Protein extracts were obtained as in Ausbel (1998), subjected to SDS-Page (NuPAGE- Invitrogen®) and probed with anti-MYC (CLONTECH, 1:5000) and anti-HA (SIGMA, 1:5000) antibodies. The expression level of the fusion proteins was then quantified with Image J software (available at http://rsb.info.nih.gov/ij; developed by Wayne Rasband, National Institutes of Health).

**
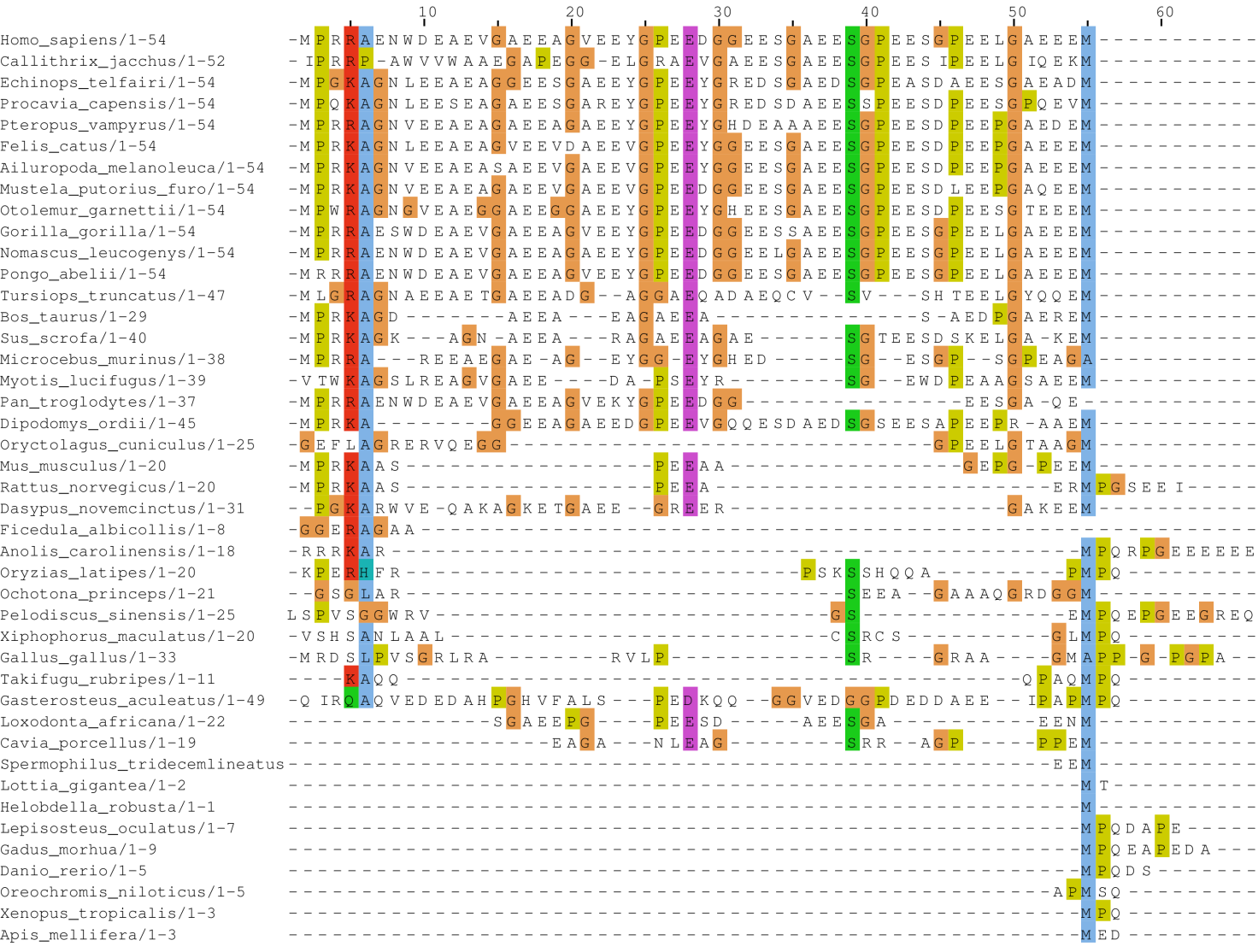
**

**Figure S2. pVHL N-terminus multiple sequence alignment for available species.**
